# NPHS2 gene polymorphism aggravates renal damage caused by focal segmental glomerulosclerosis with COL4A3 mutation

**DOI:** 10.1042/BSR20203248

**Published:** 2021-01-05

**Authors:** Liping Sun, Xinzhou Zhang, Zhen Wang

**Affiliations:** 1 Shenzhen Key Laboratory of Renal, Department of Nephrology, Shenzhen People’s Hospital, The Second Clinical Medical College, Jinan University, Guangdong, China; 2 The First Affiliated Hospital, Southern University of Science and Technology, Shenzhen 518020, Guangdong, China

**Keywords:** Focal segmental glomerulosclerosis, Gene polymorphism, Glomerular basement membrane, Renal pathology

## Abstract

Focal segmental glomerulosclerosis (FSGS), a type of primary glomerular disease, is the leading cause of end-stage renal disease (ESRD). Several studies have revealed that certain single-gene mutations are involved in the pathogenesis of FSGS; however, the main cause of FSGS has not been fully elucidated. Homozygous mutations in the glomerular basement membrane gene can lead to early renal failure, while heterozygous carriers develop renal failure symptoms late. Here, molecular genetic analysis of clinical information collected from clinical reports and medical records was performed. Results revealed that nephrosis 2 (NPHS2) gene polymorphism aggravated renal damage in three FSGS families with heterozygous COL4A3 mutation, leading to early renal failure in index patients. Our findings suggest that COL4A3 and NPHS2 may have a synergistic effect on renal injury caused by FSGS. Further analysis of the glomerular filtration barrier could help assess the cause of kidney damage. Moreover, a detailed analysis of the glomerular basement membrane-related genes and podocyte structural proteins may help us better understand FSGS pathogenesis and provide insights into the prognosis and treatment of hereditary glomerulonephropathy.

## Introduction

At present, primary glomerular diseases are the major cause of end-stage renal disease (ESRD) in China, of which focal segmental glomerulosclerosis (FSGS) accounts for 3.2–5.8% of all cases. Meanwhile, FSGS, which is showing an increasing trend over time, accounts for 12–35% of adult nephrotic syndrome (NS). In western countries, FSGS is the most common primary glomerular disease that causes ESRD [1]. FSGS can be classified into primary, secondary, and familial FSGS (FFSGS); however, its pathogenesis remains unclear [2]. FFSGS is an autosomal hereditary disease divided into autosomal dominant and autosomal recessive forms. It has been reported that more than 18% of FSGS cases were caused by family aggregation [3]. Podocyte damage plays a key role in the pathogenesis of proteinuria in kidney diseases because podocytes are involved in the maintenance of integrity of the glomerular filtration barrier. Certain genes have been identified in patients with FSGS and NS [4,5], such as components of the glomerular basement membrane (GBM), including the α3, α4, and α5 chains of type IV collagen, encoded by COL4A3, COL4A4, and COL4A5 respectively, and the structural proteins of the podocyte slit membrane, like nephrin and podocin, encoded by NPHS1 and NPHS2, respectively. Diseases, such as thin basement membrane nephropathy (TBMN), FSGS, and Alport syndrome (AS), which are caused by COL4A3–5 gene mutations, have been termed as type IV collagen-related nephropathies [5]. In addition, recent studies have shown that renal function impairment, or even ESRD, are not uncommon in patients with type IV collagen-related nephropathy carrying a COL4A3/4 gene mutation, which appears as FSGS under optical microscopy (OM) [6]. Homozygous mutations in the NPHS1 and NPHS2 genes cause congenital NS [7], eventually leading to early ESRD.

Renal biopsy (hereinafter referred to as biopsy) showed that the GBM in such patients is characterized by uneven thickness, stratification, tearing, or worm-like changes [8]. However, patients with type IV collagen-related nephropathy carrying heterozygous mutations in COL4A3 and COL4A4 show broad and diverse clinical phenotypes, and the disease spectrum varies from TBMN with microscopic hematuria or TBMN and/or FSGS with hematuria and varying degrees of proteinuria to ARAS with progressive renal failure [9]. The typical clinical manifestations of FSGS include proteinuria and podocyte damage, which often present as NS, and cause renal failure. The initial form of FSGS varies as it can occur as a result of many causes, including podocyte maladaptation, ultrafiltration, viral infection, drug use, or circulation factors [8].

In recent years, the COL4A3, COL4A4, and COL4A5 genes have been identified as important pathogenic genes for FFSGS using next-generation sequencing. Approximately 80% of adult FSGS cases are primary (or idiopathic). Xie et al. showed that 12.5% (5/40) of all FFSGS cases in China carried COL4A3 mutations, and COL4A3 is the pathogenic gene with the highest mutation rate in Chinese FFSGS [10]. Subsequently, data from Europe also showed that COL4A3, COL4A4, and COL4A5 gene mutations were the most common pathogenic mutations in FFSGS, and female with XLAS were most often misdiagnosed as FSGS [11]. In the present study, we retrospectively analyzed the data of 11 cases from 3 families with type IV collagen-related nephropathy caused by COL4A3 mutation, including clinical manifestations, histopathological changes, gene mutations, and treatment responsiveness. Interestingly, we found that NPHS2 gene polymorphism aggravated renal damage in FSGS cases with heterozygous COL4A3 mutation, resulting in severe FSGS and leading to early renal failure. Our findings may help further understand the pathogenesis of type IV collagen-related nephropathy caused by COL4A3 mutation, and may provide a reference for clinical practice in the future.

## Materials and methods

### Patients

A retrospective study was conducted from January 2013 to December 2019 among three families who were diagnosed with type IV collagen-related nephropathy and confirmed to carry the COL4A3 gene mutation by genetic testing at the Department of Nephrology of Shenzhen People’s Hospital. All samples and patient medical history were collected after obtaining informed consent. All subjects gave their informed consent for inclusion before they participated in the study. The study was conducted in accordance with the Declaration of Helsinki, and the protocol was approved by the Ethics Committee of Shenzhen People’s Hospital (LL-KY-2019293).

### Collation and analysis of clinical data

Previous hospital records and outpatient follow-up data of the members of the three families were retrieved, collated, and further analyzed for clinical features, including age at onset, onset of disease manifestations, family history of renal disease, extra-renal manifestations, degree of proteinuria or hematuria, renal function, onset of ESRD, and current treatments. Furthermore, we re-assessed the pathological data using OM, immunofluorescence, and EM. Meantime, routine urinalysis and renal function screening were performed among family members other than patients.

### Genetic analysis

Ethylenediaminetetraacetic acid (EDTA)-treated blood samples from all participants were collected after obtaining written informed consent. Genomic DNA was extracted from the peripheral blood leukocytes. Quantification and purity analysis of DNA were performed using the NanoDrop 2000 spectrophotometer (Thermo Fisher Scientific, Wilmington, DE, U.S.A.). WES was performed for three probands from three families using the SureSelect Human All Exon Target Enrichment V6 kit, which targets ∼58 Mb of the human exonic regions [3], by Shanghai Bohao Biotechnology Co., Ltd. If pathogenic mutations in the COL4A3 gene were identified by WES, consistent with the clinical data, Sanger sequencing was performed to determine whether any of the remaining variants co-segregated with the disease phenotype in this family. The encoding regions of COL4A3, COL4A4, COL4A5, NPHS1, and NPHS2 were analyzed with primers used for unidirectional Sanger sequencing: the entire coding region and the splice junction sites of NPHS1 (transcript NM_004646.3; 29 exons) and NPHS2 (transcript NM_014625.2; 8 exons); COL4A3 (transcript NM_000091.4; 52 exons), COL4A4 (transcript NM_000092.4; 48 exons), and COL4A5 (transcript NM_033380.2; 53 exons) genes. PCR primers were designed using Premier Primer 5.0 software and synthesized by Shanghai Sangon Biotechnology. The ABI PRISM 3130 Genetic Analyzer (Life Technologies GmbH, Darmstadt, Germany) was utilized to read the sequences, and the Leiden Open Variation Database (http://databases.lovd.nl/shared/variants/COL4A) was used to determine whether the co-segregated mutation sites were included in the type IV collagen-related nephropathy gene mutation database. If a new mutation was identified, Sanger sequencing was performed to determine whether the novel mutation was present in all family members with the disease. If two or more prediction software tools (Polyphen2, SIFT, and CONDEL) suggested a mutation site, the pathogenicity of the mutation site was evaluated in combination with clinical manifestations.

## Results

### Clinical characteristics

Case 1 (II-1) as a proband, in whom a severe kidney phenotype led to the discovery that in FSGS caused by genetic GBM diseases, polymorphisms in slit diaphragm genes could aggravate renal damage. Subsequently, two other families were found to have FSGS, which was aggravated by a genetic polymorphism in the slit diaphragm.

**Figure 1:**
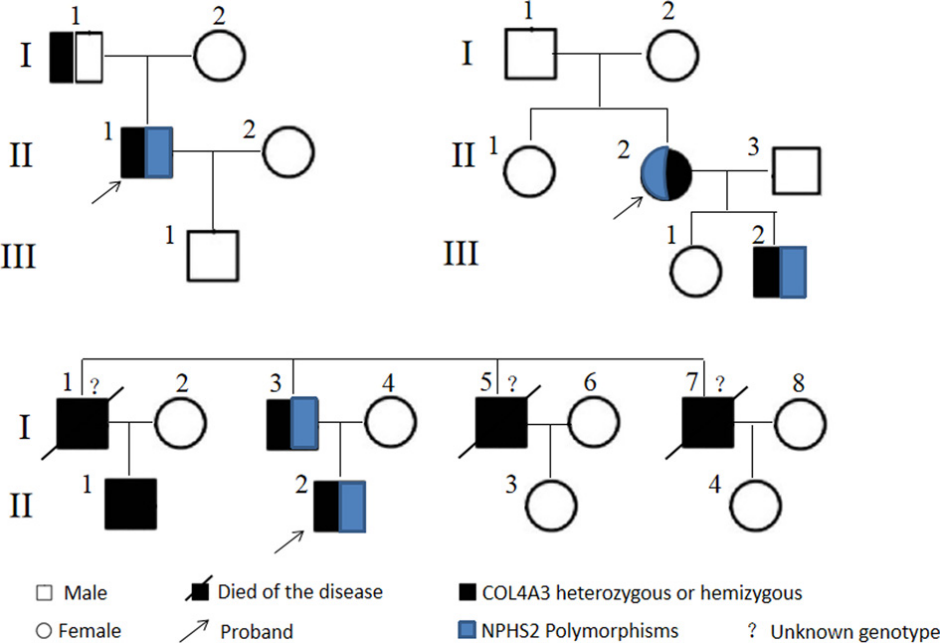
A pedigree chart of 3 Focal segmental glomerulosclerosis (FSGS) families with COL4A3 mutation

**Figure 2:**
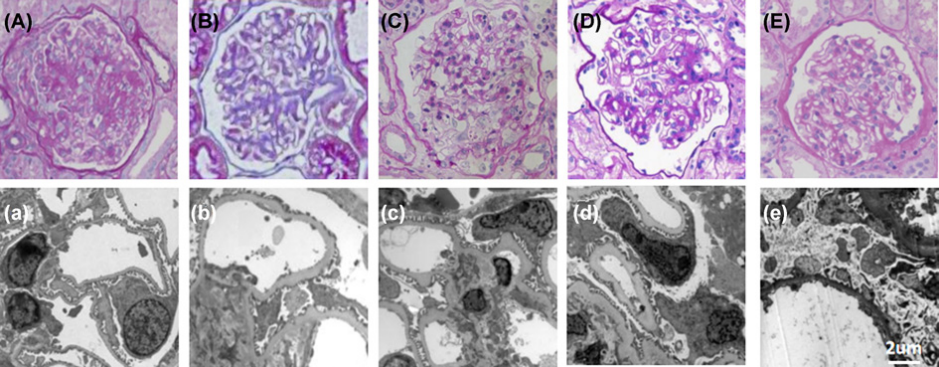
Nephropathological evaluation of the kidney biopsy of patients (**A, a**) Nephropathological evaluation of the kidney biopsy of case 1 (I-1). OM showed relatively normal glomerular and tubulointerstitial structures; electron microscopy (EM) uncovered GBM pathology with splitting and thinning; (**B, b**) For case 1 (II-1), light microscopy showed that the GBM was mildly hyperplastic and the capillary endothelial cells were moderately hyperplastic. EM uncovered that GBM pathology with endothelial injury and mesangial dilation was more obvious; (**C, c**) For case 2 (II-2), light microscopy showed that the GBM was partly severely proliferated, and the capillary endothelial cells were partly moderately and severely thickened; (**D, d**) For case 2 (III-2), light microscopy showed that the GBM was moderately thickened, the foot processes were severely fused and disappeared, and the capillary endothelial cells were severely thickened. Mesangial dilatation and endothelial damage were obvious under EM; (**E, e**) For case 3 (II-2), the tissue had only one relatively intact glomerulus and three glomerular scars. The pathology is described as non-specific, mainly sclerosis. The glomerulus showed significant peripheral fibrosis with tubular thickening of the basement membrane, which confirmed late atrophy. Magnification: (A–E) PAS, ×400, (a–e) EM, ×4000.

Patient 1 was a 31-year-old male with persistent hyperemia, proteinuria (1500 mg/l), and normal renal function. His non-cousins’ parents were healthy and had no family history of kidney disease (Figure 1). Post-infection glomerulonephritis is not included. Since ultrasonography initially suspected infection and normal renal morphology, cystoscopy was performed and hemorrhagic cystitis was excluded. Therefore, a kidney biopsy was performed. Light microscopy and immunohistochemistry (Figure 2B) showed deep FSGS and slight mesangial swelling. GBM shows diffuse division, thinning, and rupture (Figure 2B). The podocyte cells show the disappearance of the podocyte and the partial loss of the diaphragm (Figure 1). Hearing and eye assessments did not reveal any abnormalities. Nephroprotective angiotensin-converting enzyme (ACE)-inhibitor therapy inhibitors with perindopril tablets (C14202007470) treatment begins, proteinuria slowly from 1500 mg/l was reduced to less than 400 mg/l. There were no further reports of large hematuria.

Case 2 is a family with severe kidney disease, mother and son (Figure 2). The mother develops hematuria and proteinuria in her youth. She soon developed ESRD and received kidney transplant at 29 years of age. Kidney biopsy at age 21 showed FSGS, partial GBM thinning, podocytes disappeared, splitting, and lamellations. Due to the unusual nature of the severe symptoms of a girl with healthy parents, she was diagnosed with FSGS. Her son developed hematuria and progressive proteinuria in the 3 years old. Renal biopsy performed at the son was age 5, which looked relatively normal under light microscopy, but ultrastructural analysis revealed that the glomerulus cells increased, the glomeruli were split and thinned, similar to his mother’s biopsy (Figure 2C). The family refused treatment intervention. At present, the 7-year-old son has reached the third stage of chronic kidney disease (CKD).

Case 3 is a family with severe kidney disease (I-1, I-3, I-5, I-7, II-1, and II-2). The proband (II-2) was a 23-year-old man with hematuria and proteinuria and a renal biopsy showed advanced FSGS (Figure 2B,C). The biopsy material is not sufficient for ultrastructural analysis, but FSGS is highly suspected. The patient has now progressed to the fourth stage of CKD and treated with losartan potassium tablets (C14202014947). His cousin (II-1) progressed slowly with kidney disease and first had microhematuria when he was 40 years old. Several other affected family members (I-1, I-3, I-5, I-7): I-1, I-5, and I-7 died of renal failure and did not undergo genetic testing. His father (I-3) has eye involvement and started maintenance peritoneal dialysis at the age of 34.

### Characteristics of gene mutations

Genetic analysis of COL4A3 and NPHS2 using whole-exome sequencing (WES) was performed in the three families with type IV collagen-related nephropathy, and seven patients with COL4A3 mutations were identified. The pedigree chart of the three families is shown in Figure 1. As shown in Table 1, a total of three COL4A3 heterozygous mutations were identified, all of which were novel mutations. Two patients in family 1 were found to have a COL4A3 hemizygous mutation c.4145G > T (p.Gly1382Val). The proband (II-1) displayed an NPHS2 polymorphism c.725C>T (p.Ala242Val) manifested as hematuria, persistent proteinuria (1500 mg/l), and normal renal function, while the father (I-1) of the proband had no history of hematuria, proteinuria, hypertension, or CKD. Both patients in family 2 were found to have a COL4A3 heterozygous mutation c.1721C>T (p.Pro574Leu) and an NPHS2 polymorphism c.622G>A (p.Ala208Thr) manifested as hematuria, massive proteinuria and received kidney transplant at the age 29. The results of routine urinalysis of the proband’s son showed isolated microscopic hematuria, all of which were renal hematuria. Two patients in family 3 were found to have a COL4A3 heterozygous mutation c.422T>C (p.Leu141Pro), of which the proband (II-2) and the proband’s father (I-3) had a heterozygous NPHS2 polymorphism c.928G>A (p.Glu310Lys). Patient II-2 has now progressed to the fourth stage of CKD and I-3 has started maintenance peritoneal dialysis at the age of 34. His cousin (II-1) only had a COL4A3 hemizygous mutation and manifested as isolated hematuria.

**Table 1:** A summary of patient phenotypes and genotypes

Patient	Proband	Genotype COL4A3	Genotype NPHS2	Ear/Eye	Dialysis/Medication
Case 1 II-1	Yes	c.4145G > T (p.Gly1382Val)	c.725C>T (p.Ala242Val)	None	2012–today ACEi
I-1	No	c.4145G > T (p.Gly1382Val)	None	None	Refused treatment
Case 2 II-2	Yes	c.1721C>T (p.Pro574Leu)	c.622G>A (p.Ala208Thr)	None	Transplant at 29 years old
III-2	No	c.1721C>T (p.Pro574Leu)	c.622G>A (p.Ala208Thr)	None	Refused treatment
Case 3 II-2	Yes	c.422T>C (p.Leu141Pro)	c.928G>A (p.Glu310Lys)	None	2017–today ACEi
I-3	No	c.422T>C (p.Leu141Pro)	c.928G>A (p.Glu310Lys)	Blindness (2013)	Dialysis
II-1	No	c.422T>C (p.Leu141Pro)	None	None	Refused treatment

ACEi, angiotensin-converting enzyme inhibitors.

### NPHS2 polymorphism aggravates glomerular structure toward FSGS in patients with COL4A3 mutation

Further morphological analysis revealed that NPHS2 polymorphism could aggravate glomerular structural changes toward FSGS in patients with COL4A3 mutation (Figure 1). In case 1, in a male suspected of AS, genetic testing confirmed the presence of a COL4A3 mutation, which is commonly associated with microhematuria in the first few years of life and slowly progresses to microalbuminuria [12]. Therefore, the severe clinical phenotype and FSGS of the proband prompted us to further explore the NPHS1 and NPHS2 genes, which are specific for the podocyte slit diaphragm. Although both the proband and his father (Figure 2A,B) had COL4A3 mutations, the NPHS2 polymorphism in the proband exacerbated GBM pathology toward FSGS. In case 2, patient II-2 (Figure 2C) was diagnosed as having nephrotic-range FSGS with proteinuria and secondary GBM changes at a young age. Although her 6-year-old son (III-2) showed pathological features of AS with NPHS2 polymorphism, the clinical course of disease was similar to her mother (II-2) who had a heterozygous NPHS2 mutation (Figure 2D). In case 3, the renal pathology of the proband (II-2) was dominated by sclerosis (Figure 2E), which may be due to NPHS2 polymorphism. Almost no proteinuria was detected in other family members, for example, his cousins (II-1) had COL4A3 hemizygous mutations but no NPHS2 polymorphism.

## Discussion

The novel next-generation sequencing technology, which can identify most monogenic disease-causing genes, has recently been proven to screen for gene mutations associated with familial hematuria nephropathy [13]. Adult AS is easily misdiagnosed as mesangioproliferative glomerulonephritis and FSGS [14]. Therefore, more attention should be paid to the patient's family history and gene detection, which can help identify the mutation sites and reduce misdiagnosis and mistreatment. Moreover, it is very important for physicians to perform careful clinical evaluation, work closely with pathologists, and collect information about genetic mutations and polymorphisms that can affect disease. It should be noted that, in our study, relevant clinical examination of kidney histology was a basic tool and guide to genetic testing and disease evaluation. Without performing clinical and histological assessment prior to mutation analysis, physicians will not be able to understand and explain the pathology of the glomerular filtration barrier.

However, our study had certain limitations. First, phenotypic and genotypic analyses of all family members were restricted. Currently, Sanger sequencing helps to identify possible pathogenic mutations in COL4A3/4/5 and NPHS1/2, but does not detect other genetic mutations that might cause podocyte damage [15]. In spite of this, we linked the phenotypic changes to genetic alterations in most members of the three-generation family, and reported that the clinical phenotype was less severe in patients without NPHS1/2 polymorphism than in those with NPHS1/2 polymorphism; NPHS1/2 being the genes specific for the podocyte slit membrane.

Studies have shown that up to 10% of FFSGS cases are associated with autosomal COL4A3/4 mutations [16]. The findings of our study were consistent with those of previous studies. COL4A3/4 mutations are the most common cause of AS and polymorphisms in the GBM structural genes membrane aggravate FSGS [15,17]. During the progression of renal disease, podocytes play a critical role in maintaining the glomerular filtration barrier, and the dynamic regulation of the podocyte cytoskeleton is influenced by the slit membrane [18]. Podocyte dedifferentiation, podocyte effacement, and FSGS are the common features of renal disease [19]. Protection of podocytes by decreasing renal filtration pressure is an important therapeutic goal. The α3, α4, and α5 chains of type IV collagen stabilize the filtration pressure of the GBM and sense its integrity. Hence, failure to perform any one of these functions will lead to increased cytoskeleton fragility of podocytes [20]. The slit membrane is involved in the interaction between collagen receptors and the podocyte actin cytoskeleton, which may serve as a key link between the GBM and slit membrane. Since the cell–cell adhesion proteins of the slit membrane are closely linked to the podocyte cytoskeleton, any polymorphisms in the slit membrane genes may aggravate the histologic features of FSGS [21].

In summary, our study demonstrated that molecular genetic analyses of the glomerular filtration barrier can be used for the clinical assessment of FSGS. Additionally, further analysis of genes related to the structure of the slit membrane will enhance our understanding of the histopathology of FSGS. Our increasing knowledge of next-generation sequencing combined with experienced physicians and pathologists for genetic testing will help in the diagnosis, prognosis, and personalized treatment of glomerular disease.

## Data Availability

The data sets used and/or analyzed during the current study are available from the corresponding author on reasonable request.

## Abbreviations

AS: Alport syndrome
EDTA: ethylenediaminetetraacetic acid
ESRD: end-stage renal disease
FSGS: focal segmental glomerulosclerosis
GBM: glomerular basement membrane
TBMN: thin basement membrane nephropathy
WES: whole-exome sequencing
